# Medical dispatchers’ perception of the interaction with the caller during emergency calls - a qualitative study

**DOI:** 10.1186/s13049-021-00860-y

**Published:** 2021-03-09

**Authors:** Thea Palsgaard Møller, Hejdi Gamst Jensen, Søren Viereck, Freddy Lippert, Doris Østergaaard

**Affiliations:** 1 Copenhagen Emergency Medical Services, University of Copenhagen, Telegrafvej 5, 2750 Copenhagen, Denmark; 2 Clinical Research Center, Hvidovre Hospital, University of Copenhagen, Kettegård Alle 30, 2650 Copenhagen, Denmark; 3 Copenhagen Academy for Medical Education and Simulation, University of Copenhagen, Borgmester Ib Juulsvej 1, 2700 Copenhagen, Denmark

**Keywords:** Emergency medical services, Emergency call handling, Medical dispatch, Triage

## Abstract

**Background:**

Medical dispatching is a highly complex procedure and has an impact upon patient outcome. It includes call-taking and triage, prioritization of resources and the provision of guidance and instructions to callers. Whilst emergency medical dispatchers play a key role in the process, their perception of the process is rarely reported. We explored medical dispatchers’ perception of the interaction with the caller during emergency calls. Secondly, we aimed to develop a model for emergency call handling based on these findings.

**Methods:**

To provide an in-depth understanding of the dispatching process, an explorative qualitative interview study was designed. A grounded theory design and thematic analysis were applied.

**Results:**

A total of 5 paramedics and 6 registered nurses were interviewed. The emerging themes derived from dispatchers’ perception of the emergency call process were related to both the callers and the medical dispatchers themselves, from which four and three themes were identified, respectively. Dispatchers reported that for callers, the motive for calling, the situation, the perception and presentation of the problem was influencing factors. For the dispatchers the expertise, teamwork and organization influenced the process. Based on the medical dispatchers´ perception, a model of the workflow and interaction between the caller and the dispatcher was developed based on themes related to the caller and the dispatcher.

**Conclusions:**

According to medical dispatchers, the callers seem to lack knowledge about best utilization of the emergency number and the medical dispatching process, which can be improved by public awareness campaigns and incorporating information into first aid courses. For medical dispatchers the most potent modifiable factors were based upon the continuous professional development of the medical dispatchers and the system that supports them. The model of call handling underlines the complexity of medical dispatching that embraces the context of the call beyond clinical presentation of the problem.

**Supplementary Information:**

The online version contains supplementary material available at 10.1186/s13049-021-00860-y.

## Background

Emergency medical dispatching is a highly complex procedure which performance is known to impact on patient outcome [1–3]. Dispatching includes call-taking, triage, prioritizing pre-hospital resources and provision of guidance and instructions to callers. In its most time critical form, emergency call handling includes recognizing out-of-hospital cardiac arrest (OHCA), provision of dispatcher assisted cardiopulmonary resuscitation and referral to an automated external defibrillator with simultaneous and rapid dispatching of basic and advanced life support units. The importance of this process is emphasized in the recent guidelines for cardio-pulmonary resuscitation [4] and the new Global Resuscitation Alliance [5] where the interaction between the medical dispatcher and bystander, and the use of an automated external defibrillator is acknowledged as key elements for improving outcome for OHCA patients.

Medical dispatchers are gatekeepers for emergency care and emergent hospital admissions and play an essential role in terms of helping citizens with provision of first aid in hyperacute situations. Due to an increasing demand for ambulance services and limited resources, this role is even more important [6]. Assigning the appropriate resources to emergency calls is challenging. Increasingly, interest is focused on the emergency medical services (EMS) system’s ability to identify critical conditions and provide appropriate triage [7–9]. Identification of less urgent conditions has also proved important but challenging in a study where medical dispatchers categorization of emergency calls with less urgent conditions as either a specific diagnosis as opposed to an unclear problem was associated with higher mortality [10]. However, research regarding modifiable factors in emergency call handling and possibilities for improvement of performance is sparse. A study used closed circuit television recordings of actual cardiac arrests combined with emergency call recordings to explore the interaction between bystander and medical dispatcher in the case of OHCA. The study identified bystanders’ and medical dispatchers’ situation awareness, communication and attitude as main factors influencing the efficiency of the calls in terms of resuscitation [11].

Whilst medical dispatchers play a key role in the prehospital patient trajectory, their perception of challenges and solutions in the handling of emergency calls is rarely reported. In this qualitative inductive study, we aimed to explore medical dispatchers’ perception of the interaction with callers during emergency calls. Secondly, we aimed to develop a model for emergency call handling based on these findings.

## Methods

### Setting

In Denmark, a single emergency phone-number (112) leads to a primary call center for acute police, fire, or medical requests. All medical calls are redirected to an emergency medical dispatch center (EMDC). This study was conducted in the Capital Region of Denmark, covering 1.75 million inhabitants [12]. The EMDC responds to emergency calls concerning approximately 105,000 incidents annually [13]. The dispatch process comprises a medical part done by medical dispatchers with call-taking, triage and guidance and a logistic part done by logistic personnel (“technical dispatchers”) who dispatches the resource based upon the medical decision.

At the EMDC, medical dispatchers prioritize the call and provide instructions to the caller until arrival of the ambulance in case of highest priority response. The medical dispatchers are paramedics or registered nurses. Paramedics work part time at the EMDC as medical dispatchers and part time as paramedics, assisting emergency physicians in the mobile critical care unit. They follow a structured curriculum for maintenance of competences (prehospital medical expertise) in the ambulance services. Nurses work full time as dispatchers. All dispatchers, nurses and paramedics, have 6 weeks of mandatory national developed education in medical dispatching. The education consists of learning modules on communication, prehospital medicine and trauma care. The learning modules are combined with periods of practical training. Subsequently there are courses that relate to new evidence and guidelines, whenever these emerge. However, there are no formal requirements for continuous professional development in dispatch centers in Denmark. The EMDC has an emergency physician in the dispatch center 24/7 for supervision and consultation.

Dispatch processes are computerized [14]. A supportive criteria-based decision tool for handling and prioritizing emergency calls is used [15]. It supports the dispatcher’s questioning, so information provided by the caller about symptoms and severity of conditions is translated into a categorization of the main complaint into 1 of 38 different categories. Furthermore, five emergency priority levels (ranging from A-E) are determined according to the perceived severity of the problem. Level A describes life threatening or potential life threatening symptoms; B comprises urgent, but not life threatening symptoms; C is non-urgent conditions requiring an ambulance; D is non-urgent conditions requiring supine patient transport; and E includes conditions requiring medical advice only. Finally, the system comes up with a suggestion for a pre-hospital response and guidance to the caller is proposed.

### Study design

To provide an in depth understanding of medical dispatchers’ perception of their interaction with callers during emergency calls and of the dispatching process, an explorative qualitative interview study was designed. A grounded theory design and thematic analysis were used. Grounded theory assumes that all knowledge is partial and subjective and that research data are affected by the people involved. Grounded theory is appropriate for research exploring processes in specific social contexts derived from the perspective of individuals in that environment. In grounded theory the researchers do not wish to validate a preexisting theory of the studied subject, but rather inductively develop an explanatory theory or organizing concept, grounded in data, enabling understanding of the studied process [16, 17].

### Sampling and data collection

A purposive sample strategy was used to obtain information from a sample of registered nurses and paramedics. The sample was balanced in terms of professional background and later confirmed when no new subthemes emerged from interviews with either of the professions. Participants were recruited by the emergency medical services (EMS) manager, who distributed an email invitation letter to all medical dispatchers working at the EMDC (*n* = 65). Individuals who were interested in participation were asked to contact the primary study investigator. All who contacted the primary investigator were included. We aimed at approximately four focus group interviews with 4–5 participants including paramedics and nurses working as medical dispatchers. However, for operational and logistical reasons, it was only possible to conduct two focus group interviews with three nurses and two nurses and one paramedic, respectively. The remaining interviews were conducted as single person interviews with four paramedics and one nurse. This allowed a more in-depth exploration of the preliminary themes that emerged from data from the focus group interviews. The interviews were conducted between June and September 2016 by DO, TPM, HGJ, and SV. DO is a professor in medical education, TPM and SV are medical doctors, and HGJ is a Registered Nurse and master in health science. TPM, SV and HGJ were at the study time employed at the EMS Copenhagen as researchers. All authors have experience in qualitative and quantitative research within EMS. The study participants know the researchers peripheral due to their common affiliation to the Copenhagen EMS.

Data collection and analysis were performed in iterations with interview followed by coding and discussion in the research group. Semi-structured interviews were conducted using an interview guide (Table 1) created on the basis of the study aim. It was pilot tested on a medical dispatcher and adjusted accordingly. The pilot interview was not included in the analysis. The questions in the interview guide were adjusted after each interview and discussion in the research group to reflect the dispatchers’ perceptions about the call taking process. This ensured openness to new themes or subthemes. No major adjustments between focus group interviews and individual interviews were deemed necessary. However, minor adjustments, e.g. prompts on socioeconomics and interaction with the caller, were included since this was a prevalent area of interest among the interviewees. When conducting the focus group interviews, there was one interviewer and one observer present taking notes and moderating the interview and discussion. For the two first single person interviews, the same procedure was used to ensure that all emerging aspects were elaborated on.

**Table 1:** Interview guide

Interview guide*	
Frame	Main question
Introduction	• Thank you for your participation • Introduction to the project and project aims • Information about consent etc. • Demography questions
**Aim 1: dispatchers’ role in the access to emergency care**	• How do you perceive your own role in the citizens’ access to pre-hospital emergency care? • What is your role in the emergency call handling?
Preparation for aim 2: - A figure of the pre-hospital patient trajectory is shown - The emergency call process is drawn by medical dispatchers to set the scene for the interview regarding influencing factors	Notes: • The pre-hospital trajectory is shown on a timeline in which the emergency call is the key to emergency care • The emergency call has its own mini-trajectory (which is central in this project). The emergency call process is drawn by the medical dispatcher and the dissecting of the process is facilitated by interviewers
**Aim 2: factors influencing the handling of emergency calls**	• After dissecting the emergency call process, what challenges do you see, based on your experience, in the different elements in relation to handling the emergency call? • What facilitates the process?

* The initial interview guide is shown. After each interview and preliminary coding, the interview guide was adjusted in order to cover knowledge gaps

### Data analysis

Interviews were audio recorded and transcribed verbatim. Open, complete, and inductive coding was carried out through all interviews by two authors (TPM and HGJ). We positioned ourselves between the data corpus and our theoretical knowledge abductively and focused our data collection on our emerging central concepts [18]. We used our experience as researchers at the dispatch center and the existing literature as background for our primary questions yet acknowledging that this only yields plausible conclusions. Therefore, we were all the time open to new themes to dismiss or verify our preconceptions and add new knowledge. The data analysis was guided by thematic analysis though keeping the grounded theory approach towards a more focused development of data collection. Thematic analysis was considered appropriate here because this analysis is considered atheoretical. The six phase guide suggested by Braun and Clarke was followed throughout the study [19]. We first searched for meaningful units in the verbatim transcriptions. Hereafter, we paraphrased the meaning units and these were ordered in subthemes and themes by a systematic abstraction of the paraphrased meaning units in groups followed by the making if a condensate maintaining, as far as possible, the original terminology applied by the participants. Authentic illustrative quotations were also identified. In the final step we verified that the results were representative of the interviews. Internal validation was carried out during the interviews by repeating and checking the correct meaning of the interviews. Data collection continued until saturation. Data saturation was guided by Glaser’s definition of data saturation, that is where no new data is found within a particular theme [20]. To confirm the data saturation, we sought to disprove the themes with additional data from the last single person interviews. Finally, we developed a model based on the themes from the analysis.

The model was formed as a logical composition of the themes after thorough discussion and reaching consensus among the researchers. Analysis was supported by investigator triangulation and disagreements were resolved by discussion until consensus. NVivo 10 was used for the coding process. The results are reported according to the COREQ criteria [21].

## Results

### Participants and themes

Two focus groups and five single interviews were held at the dispatch center with a total of 11 participants. Among those, five were paramedics (all men) and six were registered nurses (all women). Baseline characteristics are shown in Table 2. The mean length of the interviews was 69 min (range 54–86 min). The themes that emerged regarding medical dispatchers’ perception of the emergency call process related to the caller (four themes and nine sub-themes) and the dispatcher (three themes and eight sub-themes).

**Table 2:** baseline characteristics of participants

Baseline characteristics of participants	
Age, median (range)	51 (40–60)
**Registered Nurses, n (%)**	6 (55)
Experience as nurse in years, median (range)	25 (21–29)
Experience as dispatcher in months, median (range)	54 (17–66)
**Paramedics, n (%)**	5 (45)
Experience as paramedic in years, median (range)	30 (19–33)
Experience as dispatcher in months, median (range)	36 (7–54)

### Dispatchers’ perception of caller related factors influencing emergency call handling

Four themes emerged regarding caller related factors influencing emergency call handling as perceived by the medical dispatchers. Themes and illustrating citations are presented as supplemental material.

#### Motive for calling

The dispatchers described that whilst some people were perceived calling only due to the presence of an emergency, others seemed to call due to more internal causes such as despair or lack of ability to take care of themselves. Also, lack of availability of other services or long waiting times for medical help could be a motive. It was also reported by the dispatchers that the threshold for calling seemed to be influenced by callers’ cultural and individual perceptions of when to call for help and how to use the system. Thus, some individuals called a long time after the onset of symptoms despite what seemed to be a serious condition, whereas others were reported calling very early with less severe symptoms. Callers were by dispatchers perceived as lacking knowledge about non-acute diseases and first aid, resulting in many potentially avoidable calls.

#### Situation

This theme included the circumstances of the call and the caller characteristics according to the dispatchers’ experiences*.* The circumstance related to the callers’ motive for contact (e.g. a medical cause or accident), several simultaneous calls and repeated calls. Furthermore it was reported that the caller’s distance from the patient affected the dispatcher’s ability to assess the patient. Finally, the dispatchers found that getting information directly from the patient increased the quality of the data on which the decision of response was made, as opposed to second hand information provided from relatives or bystanders. The demographic characteristics and socioeconomic position of the caller were reported to potentially affect their expectations of emergency care, and their ability to explain the situation. The dispatchers stated that the callers’ professional background was of importance; healthcare professionals were described as having higher expectations compared to non-healthcare professionals. The mental state and whether or not the caller was sober or intoxicated with drugs or alcohol was also deemed important since intoxication may mask other symptoms. Finally, the callers’ knowledge about the emergency dispatch system was described as affecting the initial dialogue due to a discrepancy in expectations.

#### Callers’ perception of problem

Callers were by dispatchers described as presenting themselves with a range of emotional states, from being in distress to being calm and focused, often affecting emergency call handling. In addition, callers’ perception of severity and urgency of a given problem differs according the dispatchers. Callers being intoxicated was also a factor within this theme. Another reported issue was the callers’ willingness to assess the patient; sometimes a clear barrier for the caller in making a close assessment of the patient was described, decreasing the dispatcher’s ability to visualize the situation.

#### Presentation of the problem

According to the interviewed dispatchers the callers’ vocal utterance, use of words and language proficiency sometimes affected the quality of the information handed over to the dispatcher and this could affect the level of details in the questions asked by the dispatcher. Terminology was another mentioned important concern due to different terms used for the same symptom or sign. The callers’ attitude were also reported influencing the process - whilst some were described humble in their description, others seem to exaggerate the story.

### Dispatchers’ perception of dispatcher related factors influencing emergency call handling

Three themes emerged relating to the medical dispatcher related factors influencing call handling as perceived by the interviewed medical dispatchers. Themes and illustrating citations are presented in Table 3.

**Table 3:** Medical dispatchers’ perception of dispatcher related factors influencing emergency call handling

Medical dispatcher related factors influencing emergency call handling			
**Example of code and meaning unit**	**Paraphrase**	**Subtheme**	**Theme**
*Using the experience to assess symptoms* [FG1] “If we take someone with chest pain. Many of us have been working in an emergency department for many, many years. Then you have seen all these. Somehow you can imagine a lot of situations, when you have seen so many things”	Registered nurses use their experience from emergency departments to assess symptoms	Experience	Expertise
*Using the experience to assess the situation* [FG2] “when you’ve worked in an ambulance for many years, and have seen people’s homes and seen how people live. I create a picture, that’ what I do. The same thing happens in road traffic accidents. Then I work with the information I get and then I can imagine, that’s the way I solve the problem”	Paramedics use their experience from the ambulance to evaluate the situation		
*Lack of updated knowledge* [FG2] “The Index helps me because I need to assess the symptoms. But sometimes I need to relate to what kind of hospital treatment the patient has had. Then it is difficult to know whether the situation is urgent or not (…) I would like some education in certain areas, right”	Besides Danish Index for Emergency Care, the dispatchers need knowledge about new treatments	Medical knowledge	
*Follow up on calls* [SI3] “sometimes I get the physician in charge to look up the patient in the record. I would like to have access to that. You use it to see if the ambulance personnel’s assessment of the patient, the measured vital signs, the ECG – if it matches the picture you created in your own head. I learn something from that. It’s continuous learning”	The dispatchers increase competence by following up on their calls	Continuous professional development	
*Paramedics and nurses as colleagues* [FG2] [Paramedic] “I think we use each other’s knowledge.” [Registered nurse] “yes, I think we use each other’s knowledge and experience in certain areas. And it is important, you have to do it, you have to use your colleagues, when you are uncertain”	Paramedics and nurses use each other’s experience mutually	Internal collaboration	Teamwork
*Physician in charge* [SI1] “all of us at the dispatch center need information about whether something is happing (in town). So people know. So people at the emergency hotline know. What is happening when a lot of people start to call. Because they do, if they are worried. Or if they have seen something distant, but something that is part of something large (…) They could just say “this and that are happening right now.” Just so we are warned about people calling about it”	The physician in charge should inform the dispatchers about larger incidents so they are prepared for receiving a lot of calls about it		
*Collaboration with ambulance personnel* [SI3] “Sometimes we contact the ambulance, if we’ve dispatched an ambulance to something that sounded urgent. And then you can see that the ambulance leaves the scene without the patient. Then okay, why does this not match my perception of the problem? Then we talk about it and I think that the ambulance personnel like it as well, because then we are more like a team, and then we are not just people that forces them to do things”	Dispatchers and ambulance personnel experience the case differently and it is valuable to discuss cases together afterwards	External collaboration	
*Response time goals* [SI1] “If we can’t live up to the response time goals, and it takes very long time for the ambulance to arrive, then the logistic personnel have to warn us. And then I have to call back to the caller to ask if they are okay or if the situation has worsened”	If the waiting time is long, the dispatchers have the responsibility to reassess the situation by calling back to the caller	Operations management	Organization
*Management* [SI2] “the management has to define the standard for what quality the company wants to deliver, and what is meant by good quality. And then it needs to be communicated to the staff, so the staff can get a common understanding of the goal, and somehow try to live up to it.”	The dispatchers need an understanding of the wanted performance level, so they know what to strive for		
*Supportive decision tool too detailed* [SI3] “it may cause fixation error. And I’ve also talked about it with the ambulance staff. That it is a problem that the information (that is handed over from the dispatch center to the ambulance) is so detailed”	The tool contains too much detail that may increase the risk for fixation error	Supportive decision tool	
*To take demography into account* [SI4] “If I should adhere to Danish Index for Emergency care accurately, then there is no difference between being 21 or 55 and having chest pain. (…) I can ask the guy at 21 about what he has been doing. And his answer could be that he has been doing workout – then it’s probably muscle soreness, but I have to send an ambulance according to Danish Index”	The tool does not take into account that the age of the patient influence disease occurrence		
*Gathering the team* [FG1] “it is the management’s responsibility to gather the team. With staff meetings and course days where they teach specific themes. And make sure that people are updated. And that the wanted competence exists. Say “this is what we want, and we will make sure that you have the competences.” Then you will do it”	The management are responsible for gathering the team	Culture in organization	
*Common understanding* [SI4] “we need a common understanding, a common goal, a common work process. It doesn’t have to be that formal. I sometimes joke with “I think I am doing good, I’ve been here for a long time and I haven’t been fired yet. (…). I assume that my level is good, because I haven’t heard the opposite””	The dispatchers need to know the defined performance level to improve their competence		
*Culture at dispatch center* [SI4] “we, at the dispatch center, have to have the same approach. That when people call, then it’s because they need help (…). We are at the same team and we have different responsibilities and we have to make things work out, because then we end up with a very good product”	Employees at the dispatch center need an understanding the common goal of helping the patients		

(…) citation is shortened; [SI]: single interview; [FG]: focus group; Text in italic: code

#### Expertise

The experience as medical dispatchers influenced the performance in terms of pattern recognition during emergency calls. Also the experience as registered nurses or paramedics played a role. Whereas the registered nurses typically created a picture of the situation based on what they had seen on a large spectrum of patients in emergency departments, paramedics used their knowledge from their daily work in the ambulance services. The nurses reported a need for more knowledge in order to clinically contextualize the information provided by callers. Paramedics indicated that they update their medical knowledge continuously as part of their curriculum. Both professional groups reported that regular visits to each other’s specialties could increase their medical knowledge and optimize their ability to visualize the situation. The continuous professional development was also mentioned as important in terms of developing and maintaining competencies. This could according to the dispatchers be through simulation-based training. Feedback with use of audio recordings was another reported option, increasing knowledge and creating reflection on own competence. However, it was reported essential that the facilitator of the feedback was a person who knew the context of emergency call handling and the organization’s desired performance standards.

#### Teamwork

In general, a need for a higher degree of “team feeling” with internal and external partners was reported. Internally, the collaboration between medical dispatchers was important during emergency calls, in terms of coordinating the resources for the incidents, and for utilizing collective expertise. The collaboration with logistical personnel was sometimes characterized by a lack of understanding of each other’s responsibilities. Similarly, it was reported that external collaboration would benefit from a better shared understanding of the different responsibilities held by each role; collaboration between medical dispatchers and ambulance personnel on scene was sometimes problematic, e.g. if the dispatcher prioritized an emergency ambulance for an uncertain incident that was perceived urgent during the emergency call, but was found to be non-urgent upon ambulance arrival.

#### Organization

This theme describes the system in which the dispatchers operate. Medical dispatchers reported working in an environment with many simultaneous tasks while at the same time trying to reach response time goals. This can affect the time spend on the individual calls and the possibility of follow-up. The physical distance to the physician in charge and the logistic personnel could affect their ability to communicate and coordinate directly, which contrasts communicating by electronic means only.

The perception of the supportive decision tool was diverse. It was described as a good support - especially in non-emergency situations. Most dispatchers used it to structure the interrogation and priority, even though their medical knowledge and experience was most important in their decision-making process. On the contrary, it was stated that too many categories are included, while important ones are missing. The category “unclear problem”, which counts for a large proportion of calls, was described as being used in the case of a lack of other appropriate categories or the presence of multiple conditions.

Finally, a general perception was that the management plays a central role in gathering the employees as a team. They are responsible for communicating the desired performance level and providing the framework for an understanding of the common goal of providing care for patients.

### A model of medical emergency call handling based on dispatchers’ perceptions

**Fig. 1:**
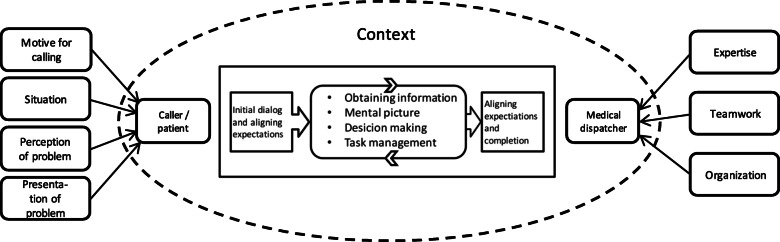
Model of emergency call handling perceived by and within the context of the emergency medical dispatcher

Medical emergency call handling was from the dispatchers’ perspective described as a procedure involving dialogue and interaction between caller and dispatcher. The core of the interaction was described as a process starting with a dialogue and an alignment of expectations, as well as reassurance of the caller if needed. This is followed by an iterative process in which dispatchers obtain information, create a mental picture, make a decision and manage the task in terms of providing instructions or an ambulance response. The interaction ends with completion of the call, optimally with another alignment of expectations. The process was described as being influenced by the context in which the caller and the dispatcher exist. A model of the workflow and interaction between caller and dispatcher is presented in Fig. 1.

## Discussion

In summary the medical dispatchers described the emergency call handling as a dialogue and an interaction between the caller and the dispatcher, influenced by their individual contexts. As perceived by the dispatcher, the motive for calling, the situation, the perception and presentation of the problem were influencing factors related to the caller. For the dispatchers, the expertise, teamwork and organization influenced the process.

Our findings of caller-related influencing factors are supported by the study by Linderoth et al., who analyzed video recordings and emergency calls regarding actual OHCA’s and found several caller-related factors influencing resuscitation performance, including the distance to the victim, the callers’ provision of information to the dispatcher and the emotional state of the caller [11]. The concept of our results could potentially improve situations like this by helping dispatchers being aware of existing barriers in the interaction with the caller and how to address them. Another recent study analyzed emergency call recordings regarding various conditions and found paradoxes related to the caller, a lack of available information, and no primary problem as important barriers [22]. Caller-related barriers are modifiable to some extent, for example public awareness campaigns could increase citizens’ knowledge about how and when to use the system. Implementation of the concept of the “first resuscitation team” in first aid courses would be another initiative, improving the interaction between caller and dispatcher [11]. However, the most potent modifiable factors are based upon the competencies of the medical dispatchers and the system supporting them.

Emergency call handling is comparable to critical decision making in other clinical settings where initial assessment is followed by decision making and subsequent task management [23]. The dispatchers´ situation awareness and decision making are very important in order to be able to provide the most appropriate guidance and response for the caller and the person in need for help. However, what makes emergency call handling particular challenging is that the assessment of the patient and their surroundings occurs without visual cues. The dispatchers’ situation awareness and hence their decision-making skills depends upon their ability to obtain information and relies therefore on their communication skills [24–26]. Non-technical skills in terms of “the cognitive, social and personal resource skills that complement technical skills” have proven to be important for health care professionals in hospital teams [27, 28]. We speculate that these skills are important for the dispatcher – caller team as well. A study of decision-making strategies for telephone triage in Emergency Medical Services showed that in cases which were not presented with a text-book description of a high urgency situation, the decisions of response made by the dispatchers were more inaccurate and lacked a connection between professional knowledge and action. Moreover, when it came to moderate-and low urgency conditions alternative decision strategies were used [29]. The view on intuitive expertise as a remarkable skill yet an error prone feature is agreed upon by prominent researchers within the field of decision-making [30]. A mental forcing strategy to overcome the mental shortcuts applied in alternative decision-making strategies is warranted, especially in emergency medicine where the dispatcher is deprived one or more of the sensory inputs [31].

The model of call handling underlines the complexity of medical dispatching that embraces the context of the call beyond clinical presentation of the problem. It is essential for managers and stakeholders to know the employees’ perceptions in the planning of professional development and specifically in planning of the curriculum and courses at the dispatch center. It is essential to acknowledge that medical dispatchers’ attitudes and preconceptions may affect their work process and decision making and therefore training should include communication and good questioning techniques besides flowcharts and the newest medical knowledge.

Medical dispatchers reported that performance differed depending on their professional background as either registered nurses or as paramedics, but importantly their collaboration complemented emergency call handling. Both groups expressed a need for continuous education and training to improve and maintain skills in emergency call handling. This finding is in line with results from previous studies [25, 32]. Our study indicates that training in non-technical skills is as important as training in medical expertise skills. Training programs should include being able to “understand the callers’ intension, how to ask the right questions and interpret the answers”. The literature on non-technical skills´ training indicate that it should be interactive, related to a given context and involving the team [28]. This could be through formal training programs using interactive methods such as roleplay and simulation-based training followed by debriefing. Simulation-based training increases performance for internal medicine residents [33]. Furthermore, it is associated with better patient outcomes [34, 35]. Feedback seems to be one of the most important factors for learning [36] and could be used more frequently also for more senior staff. The audio recordings of previous emergency calls represent a source for providing peer-to-peer feedback. The peer feedback could be planned as inter-professional (nurse – paramedic) as these two professions seems to have different experiences and expertise, which might improve individual learning. Moreover, their common experiences would increase mutual respect and confidence and develop team psychological safety [37].

### Discussion of the methods used and future perspectives

One of the strengths of this study is the sample with a balance between registered nurses and paramedics, even though the results could be biased by the fact that we may have recruited the most dedicated dispatchers in sampling by email invitation. Another strength is the triangulation of researchers participating in the data collection, analysis and interpretation of results. The authors of this paper all have professional health care background, and the main author had at the time of the conduction of the study worked at the EMS as researcher for three years. This is considered a strength as the construction of the model was a mutual interpretation from both emergency call handlers and the researchers’ in-hospital experience and experience within the EMS context. Moreover, the mix of focus-group interviews alongside in-depth interviews provided a very extensive data collection, which increases the internal validity. Other dispatch centers use the same supportive decision tool, and some employ registered nurse and paramedics in line with our system. However, there are differences in organization of systems. Nonetheless we still believe that our main findings are generalizable to other settings, dealing with the same challenges in medical dispatching. We explored medical dispatchers’ perception of the medical dispatching process as it is important for managers in dispatch centers to understand the employees’ perception of the work process. This supports the planning of continuous professional development and development of educational interventions aiming at optimizing the emergency call. However this might not reflect the callers’ perception, which could be another study of interest to further refine the results and add knowledge to a goal of a higher level of shared understanding between caller and medical dispatcher.

## Conclusions

Based on the medical dispatchers´ perception, a model of the workflow and interaction between the caller and the dispatcher was developed based on themes related to the caller and the dispatcher. The motive for calling, the situation, the perception and presentation of the problem were influencing factors related to callers, who seem to lack knowledge about the system and the medical dispatching process, as perceived by dispatchers. This can be improved by public awareness campaigns and incorporation information into first aid courses. For the medical dispatchers, the expertise, teamwork and organization were important factors, which are modifiable through continuous professional development and optimization of the system that supports them.

## Supplementary Information

**Additional file 1: **Supplemental material. Medical dispatchers’ perception of caller related factors influencing the emergency call handling.

## Data Availability

Not applicable.

## Abbreviations

OHCA: out of hospital cardiac arrest
EMS: emergency medical services
EMDC: emergency medical dispatch center
