# Supplementary material for: Medical dispatchers’ perception of the interaction with the caller during emergency calls - a qualitative study
Source: Scand J Trauma Resusc Emerg Med. 2021 Mar 9;29:45. doi: 10.1186/s13049-021-00860-y (PMC7941984; doi:10.1186/s13049-021-00860-y)
Supplement: Supplementary file 1 — Additional file 1: Supplemental material. Medical dispatchers’ perception of caller related factors influencing the emergency call handling. [file 13049_2021_860_MOESM1_ESM.docx]

**Supplemental material:** Medical dispatchers’ perception of caller related factors influencing the emergency call handling

| **Caller related factors influencing the emergency call handling** | | | |
| --- | --- | --- | --- |
| **Example of code and citation** | **Paraphrase /condensate** | **Subtheme** | **Theme** |
| *Ability to take care of yourself*  [SI1] “If ms xx aged 86 has fallen and her arm is broken, then I think it is reasonable to send an ambulance (…). I don’t think it is reasonable because you’ve hurt yourself, when going out, and now you cannot manage to take a taxi. If you’re awake and able to get into a car” | Allocation of resources also depends on citizens ability to take care of themselves | Ability to take care of themselves | Motive for calling |
| *Threshold*  [SI1] ”there are different perceptions of when to use this system and when you think an ambulance is needed. And which help you think ambulance personnel should provide. There’s an alignment of expectations there ” | The threshold for calling differs among citizens | Threshold for calling |  |
| *Waiting time at other services*  [SI3] “there are people who say ”there was waiting time at the medical helpline (…) and then they dial 112” | A motive for calling can be waiting time at other health services | Availability of other services |  |
| *Callers calling more than once*  [SI4] “When people keep calling, then maybe there is something we missed the first time or the situation has developed (…) at that time my threshold for sending an ambulance has changed even though I sometimes have to discuss with my colleagues” | When several calls are made for one incident the situation may have developed | Circumstances | Situation |
| *The caller being the patient herself or a bystander*  [SI1] ”often there are many callers. If a biker has fallen and someone has seen it, well then they call, right? But it is not implicit that he or she needs it. So if you ask to talk to the patient, it is the smartest I would say" | It is more valuable to talk to the patient than a bystander |  |  |
| *Proximity to the patient*  [FG1] (dispatcher mimics a situation where a caller from a window sees a person falling on the street) “(dispatcher): “is there anyone you can take with you? you have to assess whether the patient is breathing – (caller): “he doesn’t, just send that ambulance” (…) (dispatcher): “you have to go down. If there is no one down there (…) I will stay with you if you are scared” | In case of the caller being too far away from the patient, it is difficult to obtain information |  |  |
| *Demography*  [SI1] ”There are many older people, who want to tell you about all their hospitalizations to make sure that you have all the information, right? And that makes sense, but it takes time until you actually get to the point, right” | Older people often report a longer history | Characteristics of caller |  |
| *Intoxicated*  [FG1] “it is an art just to clarify what the problem really is (…). they might have been drinking, that we can handle, initially without an ambulance. But there may be more to it. We cannot just say that they have been drinking. We have to find out if they have diabetes as well” | Intoxicated people can have underlying conditions that dispatchers needs to be aware of |  |  |
| *Knowledge about available resources*  [FG2] ””just send an ambulance.” there is no understanding of that there is not enough ambulances in the inner city. You sometimes have to explain why we cannot allocate an ambulance to the case. But for the caller, maybe it’s the first time they ever call 112 and they think it’s very important | Citizens are not aware of the limited resources |  |  |
| *Knowledge about what to expect*  [SI4] “I think there is a lack of information about – when you call, the dispatchers do not sit there to annoy you. They are there to help you. It is important that you are ready to provide some information. What number you call from, who are you, where are you, what has happened and how many patients” | People don’t know that the dispatchers ask questions to obtain information for their decision-making process |  |  |
| *Healthcare providers*  [SI1] ” physicians tend to dictate more, they want something and then things should be this way. It's just a little funny sometimes (...) one could expect a more decent dialogue, because we are actually in the same business after all, right. There is sometimes a little conflict when healthcare professionals are the callers” | Healthcare providers have often triaged the incident prior to the call. |  |  |
| *Reluctance*  [FG2] ”some people call from their apartment looking at a bench on the street, because someone is lying there. Then we have to convince them to go down and have a look. Others do not want to touch other people, then we just have to come as fast as possible | People sometimes feel reluctance, affecting dispatchers’ opportunity to assess the patient | Reaction | Callers perception of problem |
| *Intoxicated callers’ perception of problem*  [SI2] ”the drunk people just have an altered sense of reality, so to speak, than sober people (…) they sometimes live in rough environments (…) that can be a pitfall. Things that for others seem to be severe and urgent, can for them be banal and say “he was just beaten and hit his head, he just has to go home and sleep”” | Intoxicated people can have a different perception of severity and urgency than sober people | Self-evaluation of severity and urgency |  |
| *Mismatch between dispatchers’ and callers’ perception of severity*  [SI3] ”I sometimes think that there is something really wrong and send a very urgent response, and then in reality it is nothing. Other times people call and are very calm and then in reality it is really serious, that is the range we work with” | There can be a large discrepancy between the dispatchers and the caller’s perception |  |  |
| *Humble citizen*  [FG2] ”then there are the people that know something is wrong, but they won’t admit that something is wrong. Typically, it’s the older generation, they say “I don’t feel very well, but it’s not that way… I had chest pain, I have breathing difficulties, but I’m alright.” (…) You know, they don’t want to be ill (…) then you have to use your intuition. ” | Dispatchers use their intuition to assess what is the true severity of the problem | Attitude | Presentation of problem |
| *Insisting caller*  [FG1] ”if they speak rudely to me, and they won’t answer any questions. “Just send that ambulance, don’t ask all these stupid questions.” Then I’m affected, even though I really try not to. | Dispatchers can be affected by the way callers speak to them |  |  |
| *Spoken language and terminology*  [FG1] ”There is the spoken language. That is whether it is Danish or Turkish. But another thing is if someone is unconscious, then the caller says ”he is all gone,” I mean, they use other terms and expressions, then you have to find out – what does “all gone” mean? Is that unconsciousness or something else?” | Both the spoken language and terminology influence the quality of provided information | Language / terminology |  |
| (…) citation is shortened; [SI]: single interview; [FG]: focus group; Text in italic: code | | | |
